# END-OF-LIFE CARE AND DECISION-MAKING IN CHINA: HOW TO IMPROVE END-OF-LIFE CARE QUALITY?

**DOI:** 10.1093/geroni/igae098.1744

**Published:** 2024-12-31

**Authors:** Yifan Lou, Yaolin Pei, Bei Wu

**Affiliations:** Chair; Co-Chair; Discussant

## Abstract

Alongside population aging comes the challenges of providing quality end-of-life care in China, where the largest and most rapidly growing older adults reside. Mounting qualitative evidence has documented the culturally different needs, experiences, and barriers of end-of-life care and decision-making among older Chinese, but the quantitative evidence is lacking. Aligned with GSA 2024’s theme, this symposium uses national and regional data to investigate potential “fortitude factors” that can improve end-of-life qualities for older adults and reduce burden for caregivers in China. The first paper will share policy-relevant findings on the urban-rural differences in pain, place of death, and their associations with the use of pain treatment at the end of life, a cornerstone of end-of-life dignity. The second paper will turn the attention to caregivers and present a population-based study on the individual- and household-level factors that may contribute to end-of-life caregiving burden and the role of the COVID-19 pandemic. Two studies on end-of-life care decision-making with a dyadic perspective will then be presented. The third one will focus on the children’s role, considering both number of children and financial responsibilities, in deciding end-of-life treatments for their parents under the culture of filial piety, using retrospective data. The fourth one will then describe older adults’ preferences for involvement and roles in their future end-of-life care decision-making with their loved ones and doctors, using regional survey data. Lastly, our discussant, Dr. Bei Wu, will synthesize these presentations and discuss the policy and practice implications in improving “good death” in China.

